# Rotavirus Genotypes and Vaccine Effectiveness from a Sentinel, Hospital-Based, Surveillance Study for Three Consecutive Rotavirus Seasons in Lebanon

**DOI:** 10.1371/journal.pone.0161345

**Published:** 2016-08-29

**Authors:** Zainab Ali, Houda Harastani, Moza Hammadi, Lina Reslan, Soha Ghanem, Farah Hajar, Ahmad Sabra, Amjad Haidar, Adlette Inati, Mariam Rajab, Hassan Fakhouri, Bassam Ghanem, Ghassan Baasiri, Bernard Gerbaka, Hassan Zaraket, Ghassan M. Matar, Ghassan Dbaibo

**Affiliations:** 1 Department of Pediatrics and Adolescent Medicine, American University of Beirut, Faculty of Medicine, Beirut, Lebanon; 2 Center for Infectious Diseases Research, American University of Beirut, Faculty of Medicine, Beirut, Lebanon; 3 Department of Biochemistry and Molecular Genetics, American University of Beirut, Faculty of Medicine, Beirut, Lebanon; 4 Department of Pediatrics, Nini Hospital, Tripoli, Lebanon; 5 Department of Pediatrics, Makassed General Hospital, Beirut, Lebanon; 6 Department of Pediatrics, Rafic Hariri University Hospital, Beirut, Lebanon; 7 Department of Pediatrics, Nabatieh Governmental Hospital, Nabatieh, Lebanon; 8 Department of Pediatrics, Hammoud Hospital, Saida, Lebanon; 9 Department of Pediatrics, Hôtel-Dieu de France University Hospital, Beirut, Lebanon; 10 Department of Experimental Pathology, Immunology and Microbiology, Faculty of Medicine, American University of Beirut, Beirut, Lebanon; Tulane University, UNITED STATES

## Abstract

**Introduction:**

Globally, rotavirus (RV) is the leading cause of gastroenteritis (GE) in children. Longitudinal data about changes in RV genotype distribution and vaccine effectiveness (VE) are scarce. This study was conducted in Lebanon over 3 consecutive RV seasons to estimate the rate of RVGE hospitalization, identify RV genotypes, determine the seasonal and geographical variations, and calculate RV VE.

**Materials and Methods:**

This prospective, multicenter, hospital-based surveillance study was conducted between 2011 and 2013 and enrolled children (<5 years) admitted for GE. Socio-demographic and clinical data about the current episode of GE at admission were collected. Genotypes were determined from stool samples testing positive for RV by PCR.

**Results:**

Of 1,414 cases included in the final analysis, 83% were <2 years old and 55.6% were boys. Median duration of hospitalization was 4 days and 91.6% of GE cases were severe (Vesikari score ≥11). PCR testing showed that 30.3% of subjects were RV-positive of which 62.1% had fever *versus* 71.1% of RV-negative subjects (*P* = 0.001). RV was predominantly detected in the cold season from November till March (69.9%). G and P genotype pairs for all RV-positive stool specimens showed a predominance of G1P[8] in 36% (n = 154) of specimens, G9P[8] in 26.4% (n = 113), and G2P[4] in 17.8% (n = 76). RV-negative subjects were more likely to be RV-vaccinated (21%) compared to the RV-positive subjects (11.3%) (*P*<0.001), with a vaccine breakthrough rate of 18.8%. The ratio of RV1-vaccinated for each RV5-vaccinated subject was 7.8 and VE against RV disease was 68.4% (95%CI, 49.6%-80.2%).

**Conclusion:**

RV is a major cause of GE requiring hospitalization of children under 5 years of age in Lebanon. A few genotypes predominated over the three RV seasons studied. Mass RV vaccination will likely decrease the burden of hospitalization due to RV. VE is similar to what has been observed for other middle-income countries.

## Introduction

Rotavirus (RV) is the leading cause of severe gastroenteritis (GE) in infants and young children around the world [1]. Rotavirus is a double-stranded RNA virus belonging to the *Reoviridae* family. It is a complex particle with viral genome segments encoding 6 structural viral proteins (VPs), which make up the virus particles, and 6 non-structural proteins involved in viral virulence. The outer capsid is made of VP7 and VP4 proteins, the latter responsible for the capsid’s spikes. Both proteins are neutralizing antigens in the immune response to RV infection. In the early 1990’s, genotyping was introduced based on sequences of the *VP7* and *VP4* genes, and has now replaced serotyping as the preferred method of typing. P genotypes are denoted in brackets (P[8], P[4]…) whereas serotypes are denoted in parentheses, for example P(8) [2].

RV infection can range from being asymptomatic to a severe symptomatic one [3]. The main clinical symptoms of RV infections are fever, vomiting, and diarrhea in infants and young children. The outcome of the infection is affected by both viral and host factors, and the major host factor determinant is the patient’s age. The age group most susceptible to severe RV infection ranges from 3 months to 2 years. RV infection is generally asymptomatic in neonates, since they are protected by transplacental transfer of maternal antibodies [3]. As for adults, the incidence of RV infection is low and symptoms are unlikely to be severe although outbreaks in adolescents and adults have been described [3].

The first human rotaviruses were discovered in 1973 [4,5]. Soon afterwards, RV was found to be associated with 20 to 30% of severe diarrheal cases that required hospitalizations in children younger than 5 years of age worldwide [6]. Globally, it has been estimated that there are more than 114 million RV diarrheal episodes per year requiring home care with an additional 24 million clinic visits, 2.4 million hospitalizations, and more than 600,000 deaths in children younger than 5 years of age [7]. Additional epidemiological studies have estimated that by 5 years of age, one in 50 children is hospitalized and one in 205 children dies from RV-associated causes [7,8]. In Western Europe, prior to the vaccine era, RV was responsible of 230 deaths per year [9,10]. Pre-vaccine epidemiological studies also reported that 50% of cases of gastroenteritis in children younger than 5 years of age who were treated in the emergency room were caused by RV in the USA and Western Europe [9,10,11]. Consequently, one out of 150 children younger than 3 years of age would be hospitalized and 1 out of 11 would be seen as an outpatient in an emergency room for RV disease [11]. Importantly, RV is a common hospital-acquired infection where 20% of children admitted to the hospital for another cause would acquire a RV infection during their stay throughout the year [11,12].

Furthermore, the burden of the RV infection is not limited to morbidity and mortality, but is also associated with significant economic losses. The latter exceeds 1 billion US dollars per year in USA alone, prior to vaccine introduction [13].

RV is common in temperate and tropical climates, as well as in developed and less-developed social settings. Despite the policies to improve the hygiene levels in these countries, decreasing the transmission of RV is impossible because of many factors. First, rotaviruses are shed in feces in amounts up to 1010 particles per gram of stool [14]. Second, the infectious dose has been estimated to be as low as one infectious virus particle. RV is also highly resistant to commonly used disinfectants and will survive for several days on fomites [15]. RV infections vary throughout the year in many regions of the world. Nevertheless, RV disease seems to be seasonal in temperate climates, occurring in the dry and cool months and less likely in warm and humid months of the year [14,16].

No specific treatment of RV infections is available and only supportive care to prevent dehydration and its complications is provided where available. Vaccination is the only effective prevention strategy. Bovine rotavirus RIT 4237 vaccine was initially developed but was never marketed [17]. A simian RV reassortant vaccine (Rotashield^®^) was developed and marketed in August 1998. However, it was withdrawn from the market in October 1999 because of its association with a high incidence of intussusception [18]. In 2006 and 2007, two oral vaccines for RV GE (Rotateq^®^ and Rotarix^®^) were licensed in many countries. Rotateq^®^ (Rotavirus Vaccine, Live, Oral, Pentavalent, Merck Sharp & Dohme corp., USA) is a human-bovine reassortant vaccine which was found to be highly efficacious with a protection rate of 74% against diarrhea of any severity [19]. Rotarix^®^ (Rotavirus Vaccine, Live, Oral, GlaxoSmithKline Biologicals, UK) is a human G1P[8] RV vaccine. Rotarix^®^ was 85% effective against preventing severe diarrhea in a large cohort study [19,20]. Both vaccines were 100% effective against the most severe cases [19].

Epidemiological data about RV infections are scarce in Lebanon. We recently published the results of a hospital-based surveillance study in five large urban, private hospitals in North, Central and South Lebanon, conducted between April 2007 and September 2008 essentially covering one RV season [21]. Baseline data are essential to understand the burden of the disease and to make suitable recommendations to control RV infections in the country. Therefore, our current study is the second to report on the epidemiology of this disease in Lebanon but covering three consecutive RV seasons. Our main goal was to assess the rate of RV infections in a sentinel surveillance system in the Lebanese pediatric population (less than 5 years). The secondary objectives were (1) to estimate the rate of diarrheal hospitalization due to RV, (2) to determine the age and the seasonal distribution of RV-associated hospitalization, (3) to identify the most prevalent RV genotypes, (4) to determine the seasonal changes and year-to-year changes with respect to predominant RV genotypes in the target population, and (5) to assess the vaccine effectiveness (VE) of RV1 and RV5 against rotavirus disease. The results are expected to help policy makers understand the importance and magnitude of RV infections so that vaccine recommendations can be formulated accordingly.

## Material and Methods

### Study procedure

Our study was a prospective, multicenter, hospital-based surveillance in seven medical centers distributed in North, Central, and South Lebanon. It was conducted over 30 months from January 2011 through June 2013. Eligible participants were children below 5 years of age, admitted mainly for GE, to the designated centers. Infants and children hospitalized more than once were considered as new participants at each new admission. Also, a stay in the emergency department longer than 12 hours was considered a hospitalization. To be enrolled in the study, the child’s parents or guardians had to be believed by the investigators to comply with the protocol’s requirements. A written informed consent was obtained from the parents or guardians. Infants and the children whose admission diagnosis did not include GE or whose onset of GE occurred more than 12 hours after admission to the hospital (hospital-acquired infections) were excluded.

### Data collection

Data were collected by the investigators using a standardized English case report form (CRF) (S1 CRF). Information collected during hospitalization included data about age, gender, area of residence, height and weight of the patients, their past medical history and their current episode of GE at admission (body temperature, duration of diarrhea or vomiting). The severity of RV gastroenteritis was assessed using the Vesikari scale [22]. Information about any prior treatments, rehydration therapy, rotavirus vaccination, and antibiotics were also reported. Further, the date, the diagnosis at discharge and the outcome of GE (recovery, recovery with sequelae, death, transfer to another hospital, ongoing or unknown) were recorded.

### Laboratory analysis

One stool sample was collected from each patient in sterile stool sample containers preferably within four days and not later than 10 days after the onset of GE symptoms. The detection of rotavirus antigen was tested using the SD Bioline Rotavirus rapid kit (Standard Diagnostics, INC., Republic of Korea). This test was performed according to the manufacturer’s specifications.

All RV-positive samples were processed for RNA extraction and genes sequencing. Double-stranded viral RNA was extracted from 10% stool suspensions that were made by adding 0.5 g or 500 μl of the fecal sample to 5 ml of NaCl solution (0.89%). The homogenate was clarified by centrifugation at 4,000 g at 4°C for 20 minutes. The supernatant was re-centrifuged at 1,500 g at 4°C for 10 minutes. RNA was extracted from 420 μl of the clarified supernatant using the QIAamp^®^ Viral RNA Mini Kit (Qiagen, Hilden, Germany) in accordance with the manufacturer’s spin protocol. RNA was eluted in 45 μl RNase-free distilled water and stored at -20°C.

For specimens that were negative by antigen detection, every twentieth sample collected chronologically was subjected to real-time reverse-transcription polymerase chain reaction (RT-PCR) testing to confirm the absence of rotavirus. Double-stranded RNA was denatured at 97°C for 5 minutes and reverse-transcribed and amplified using the Qiagen One-Step RT-PCR Kit (Qiagen, Hilden, Germany). Previously published primers were used for the amplification of genes encoding for VP7 genes (nt: 37–932) and VP4 genes (nt: 11–887) [23]. The target region of each gene was reverse-transcribed at a temperature of 42°C for 30 minutes followed by deactivation of the reverse-transcriptase at 95°C for 15 minutes. Amplification of each gene was performed on a C1000 thermal cycler (Bio-Rad, Inc., Berkeley, California, USA) using the following cycle parameters: 30 cycles of 94°C for 30 seconds, 42°C for 30 seconds, and 72°C for 45 seconds, and final extension at 72°C for 7 minutes. Amplicons were detected by gel electrophoresis using a 1.5% agarose gel and analyzed and photographed by a gel documentation system (Gel doc XR, Bio-Rad, Berkeley, California, USA). RT-PCR products were cleaned using ExoSAP-IT^®^ according to manufacturer’s instructions (USB Corp., Cleveland, OH, USA) and used directly for sequencing. Sequencing was performed at Macrogen institution (Seoul, Republic of Korea). The sequence data were analyzed by using BioEdit v7.2.5 software. The genotype assignment for each gene was performed using the BLAST (Basic Local Alignment Search Tool) server on the GenBank database at the National Center for Biotechnology Information (NCBI) and the online RotaC v2.0 rotavirus genotyping tool (available at: http://rotac.regatools.be).

### Data analysis

The total population of children aged below 5 years of age in Lebanon was 219,150 during the study conduct, and about 21% of this population was to be served by the seven participating study centers as estimated by the Lebanese Ministry of Public Health and the Mundi Index [24]. Therefore, the total population of children in the same age category and served by the designated centers was estimated at 46,000. Moreover, the total number of GE cases admitted to the study centers were estimated as 800 per year. As a result, the incidence of GE hospitalization in the study sample was expected to be 1.74 (800 over 46,000). Based on 30-month enrollment period, 115,000 children-years were to be followed up, of whom 2,000 GE episodes were expected with all children participating in the study over a period of two and a half years. Assuming that 20 to 40% of these GE episodes would be RV-positive GE, approximately 400 to 800 RV-positive GE episodes would be possible to be captured by year. Thus, 2,000 children were targeted in this study considering that all episodes of GE hospitalizations at the participating hospitals were to be enrolled over two and a half years.

Records for participants with incomplete, insufficient or conflicting data were excluded. Sample characteristics were summarized using the mean and the standard deviation (SD) for continuous variables such as age; frequency distributions for categorical variables such as gender were presented. Incidence rates were calculated using descriptive data, along with its corresponding 95% confidence interval (CI). Season-to-season variation in RV infections and predominant rotavirus genotypes were described by geographical areas and seasons. Season 1 extended from January 2011 to August 2011, season 2 from September 2011 to August 2012, and season 3 from September 2012 to June 2013. Characteristics of the children were also compared using the chi-square test and Fisher Exact test as appropriate.

As for vaccine effectiveness, it was computed along with its 95% CI as (1-odds ratio [OR]) × 100%, by logistic regression. The logistic regression had RV-positive and RV-negative as outcome and vaccination status as explanatory variables. ORs were adjusted by geographic region and season. Patients were assumed to have the proper vaccination if they had two documented RV1 vaccination or 3 documented RV5 doses all 14 days before hospital admission.

Significance level was two-sided and set at 5%. The statistical analysis was carried out using IBM-SPSS version 21 software for Windows Release (IBM Corp. Released 2012. IBM SPSS Statistics for Windows, Version 21.0. Armonk, NY: IBM Corp.).

### Ethical considerations

The study was conducted according to Good Clinical Practice, and the 1996 version of the Declaration of Helsinki. It was also approved by the institutional review boards (IRBs) of each of the participating centers (IRB of the American University of Beirut, IRB of Nini Hospital, IRB of Makassed General Hospital, IRB of Rafic Hariri University Hospital, IRB of Nabatieh Governmental Hospital, Nabatieh, Lebanon, IRB of Hammoud Hospital, and Ethics Committee of Hôtel-Dieu de France University Hospital) before initiating the study. Information about the infants and the children whose parents or guardians accepted to participate in the study were collected after signing the written informed consent. Participants’ numbers were assigned sequentially to all children whose parents or guardians signed the consent form according to the range of the children’s numbers assigned to every center. Data collection and analysis were performed respecting the participants’ autonomy and anonymity.

## Results

### Socio-demographic and clinical characteristics of the patients diagnosed with GE

The distribution of the demographic and clinical characteristics of the children hospitalized for GE according to their RV status is displayed in Table 1. Over the 30-month surveillance program, 1,555 children were hospitalized at the seven study centers for GE and were enrolled in the study but 141 were excluded due to inability to obtain a stool sample or due to loss of the case report form during transport to the main center. The remaining 1,414 children were included in the final analysis. Of the 1,414 fecal samples tested, 30.3% (n = 428, 95%CI, 27.9%-32.7%) were RV-positive by PCR. Of the samples that were negative for RV by immunoassay and were subsequently screened by PCR, none were positive.

**Table 1:** Distribution of demographic and clinical outcomes of patients according to RV status.

Variable	Overall	RV-positive	RV-negative	*P*-value*
	(n = 1,414†)	(n = 428†)	(n = 986†)	
**Center**				0.024*
AUBMC	157 (11.1%)	44 (28.0%)	113 (72.0%)	0.030*£
Hammoud Hospital	209 (14.8%)	83 (39.7%)	126 (60.3%)	
Hôtel-Dieu de France University Hospital	5 (0.4%)	0 (0.0%)	5 (100.0%)	
Makassed Hospital	189 (13.4%)	47 (24.9%)	142 (75.1%)	
Nini Hospital	556 (39.3%)	166 (29.9%)	390 (70.1%)	
Nabatieh Hospital	212 (15.0%)	65 (30.7%)	147 (69.3%)	
Rafic Hariri University Hospital	86 (6.1%)	23 (26.7%)	63 (73.3%)	
**Age (months)**				0.984
0–2	93 (6.6%)	30 (32.3%)	63 (67.7%)	
3–6	268 (19.0%)	79 (29.5%)	189 (70.5%)	
7–12	461 (32.6%)	137 (29.7%)	324 (70.3%)	
13–24	364 (25.7%)	112 (30.8%)	252 (69.2%)	
25–60	228 (16.1%)	70 (30.7%)	158(69.3%)	
**Male gender**	786 (55.6%)	235 (29.9%)	551 (70.1%)	0.734
**Living in Lebanon**	1350 (95.5%)	413 (30.6%)	937 (69.4%)	0.252
**Vesikari score**>10	1,295 (91.6%)	398(93.0%)	898(91.1%)	0.231
**Symptoms**				
Fever	966 (68.3%)	266 (62.1%)	700 (71.1%)	0.001*
Vomiting	889 (62.9%)	327 (76.4%)	562 (57.0%)	<0.001*
Diarrhea	1,288 (91.1%)	385 (90.0%)	903 (91.7%)	0.295
**Treatments during hospitalization and at discharge**‡				
Oral rehydration	234 (16.6%)	85 (19.9%)	149 (15.1%)	0.028*
IV rehydration	1,379 (97.5%)	416(97.2%)	963(97.7%)	0.600
Antibiotics	698 (49.4%)	173(40.4%)	525(53.2%)	<0.001*
**Vaccination history**				
Yes (any type and dose)	255 (18.1%)	48(11.2%)	207 (21.0%)	<0.001
RV1 (Rotarix^®^)				
1 dose	54 (25.0%)	13 (32.5%)	41 (23%)	
2 doses	165 (75.0%)	27 (67.5%)	138 (77%)	
RV5 (Rotateq^®^)				0.973
1 dose	8 (28.5%)	2 (28.5%)	6 (28.6%)	
2 doses	3 (10.7%)	1 (14.3%)	2 (9.5%)	
3 doses	17 (60.8%)	4 (57.2%)	13 (61.9%)	
Unknown vaccine	2 (0.1%)	2 (0.5%)	0 (0.0%)	
Unvaccinated	1,157 (81.8%)	378 (88.3%)	779 (79.0%)	
**Duration of hospitalization (days)**‡				0.106
0–3	537 (38.0%)	145 (33.9%)	392 (39.8%)	
4–7	805 (56.9%)	261 (61.0%)	544 (55.2%)	
>7	72 (5.1%)	22 (5.1%)	50 (5.1%)	

^†^The sample size per column might not add to the total of that column depending on whether there are some missing data or not.

*Significant at the 5% level.

^£^*P*-value excluding Hôtel-Dieu de France University Hospital from the comparison.

^‡^Percentages are computed within each column (i.e. percentage of treatment within each group of RV+ and RV-).

Abbreviations: AUBMC = American University of Beirut Medical Center.

Overall, age-specific rates of the patients with GE rose sharply from age zero to 7 months (25.6%), peaked in the 7–12 months age group (32.6%), and subsequently dropped to (25.7%) for those aged 13–24 months and (16.1%) for those aged 25–60 months with no significant difference in age-specific rates between the RV-positive and RV-negative groups. In both groups, 83% of the patients were younger than 2 years of age, 55.6% were boys and only 4.5% were not living in Lebanon.

The median duration of hospitalization was 4 days and ranged between 1 and 73 days with vast majority of GE cases (91.6%) being severe according to Vesikari score of 11 or above. The difference in the duration of hospitalization between the RV-positive and RV-negative children was not statistically significant.

**Fig 1:**
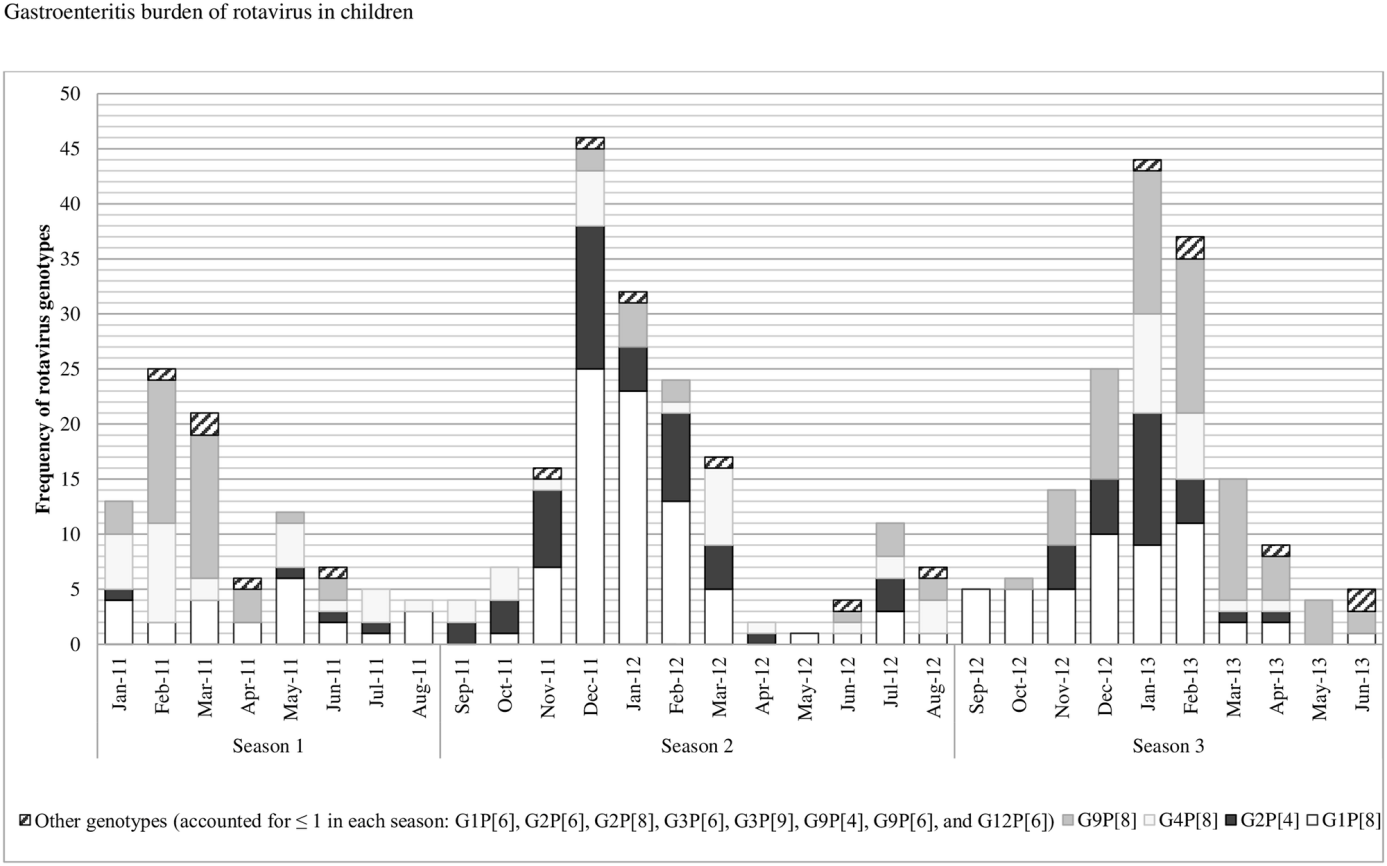
Season-to-season variation in RV infections and predominant rotavirus genotypes (in frequency) across seasons between January 2011 and June 2013 (n = 428).

Patients with RV-positive GE significantly differed from the RV-negative group by fever and vomiting, treatments during hospitalization and at discharge (oral rehydration and antibiotics), year of hospitalization, and RV season. As such, fewer RV-positive patients had fever compared to those who were RV-negative (62.1% *versus* 71.1%, *P* = 0.001); more RV-positive children (19.9%) were treated with oral rehydration while only 15.1% were offered the same treatment in the RV-negative group (*P* = 0.03) probably related to the increased incidence of vomiting in the former group (76.4% vs 57%, *P* <0.001). 53.2% of children with RV-negative GE were given antibiotics compared to 40.4% in the RV-positive group (*P*<0.001). Additionally, the incidence of RV-positive GE associated hospitalization reached its maximum (53.8%) in 2013 while RV-negative GE (75.7%) was more frequent in 2011 (*P*<0.001). Overall, total GE peaked in the month of July (12.3%). RV was encountered throughout the 30-month study period, but was predominantly detected in the cold season from November till March (69.9%) (Table 1 and Fig 1).

### RV genotyping

**Fig 2:**
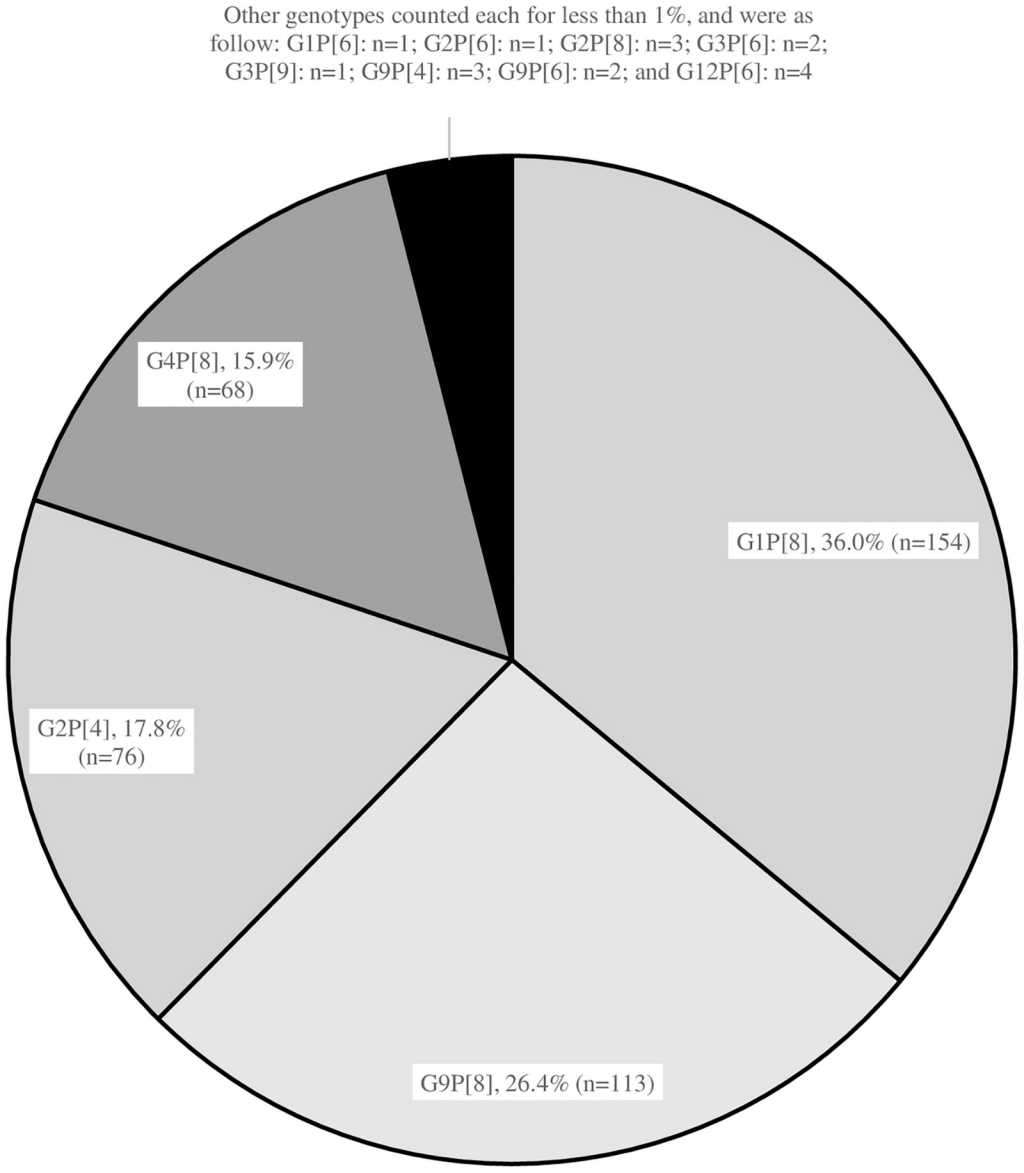
Distribution of the RV genotypes among all RV-positive specimens (n = 428).

G and P genotypes of rotavirus were determined in the 428 RV-positive stool specimens out of the total 1,414 samples (30.3%) by sequencing of the VP7 and VP4 genes, respectively. There was a predominance of G1P[8] RV accounting for 36% (n = 154) of specimens, G9P[8] RV was responsible for 26.4% (n = 113) of cases, G2P[4] for 17.8% (n = 76) and G4P[8] for 15.9% (n = 68) (Fig 2).

Furthermore, RV genotype predominance differed significantly across seasons. As such, G9P[8] was predominant in seasons 1 and 3, while G1P[8] was the major genotype in season 2 (*P*<0.001). As for G2P[4], it was more frequent in season 2 compared to seasons 1 and 3 (*P*<0.001), and G4P[8] was more encountered in seasons 1 and 2 rather than season 3 (*P* = 0.004) (Fig 1).

**Fig 3:**
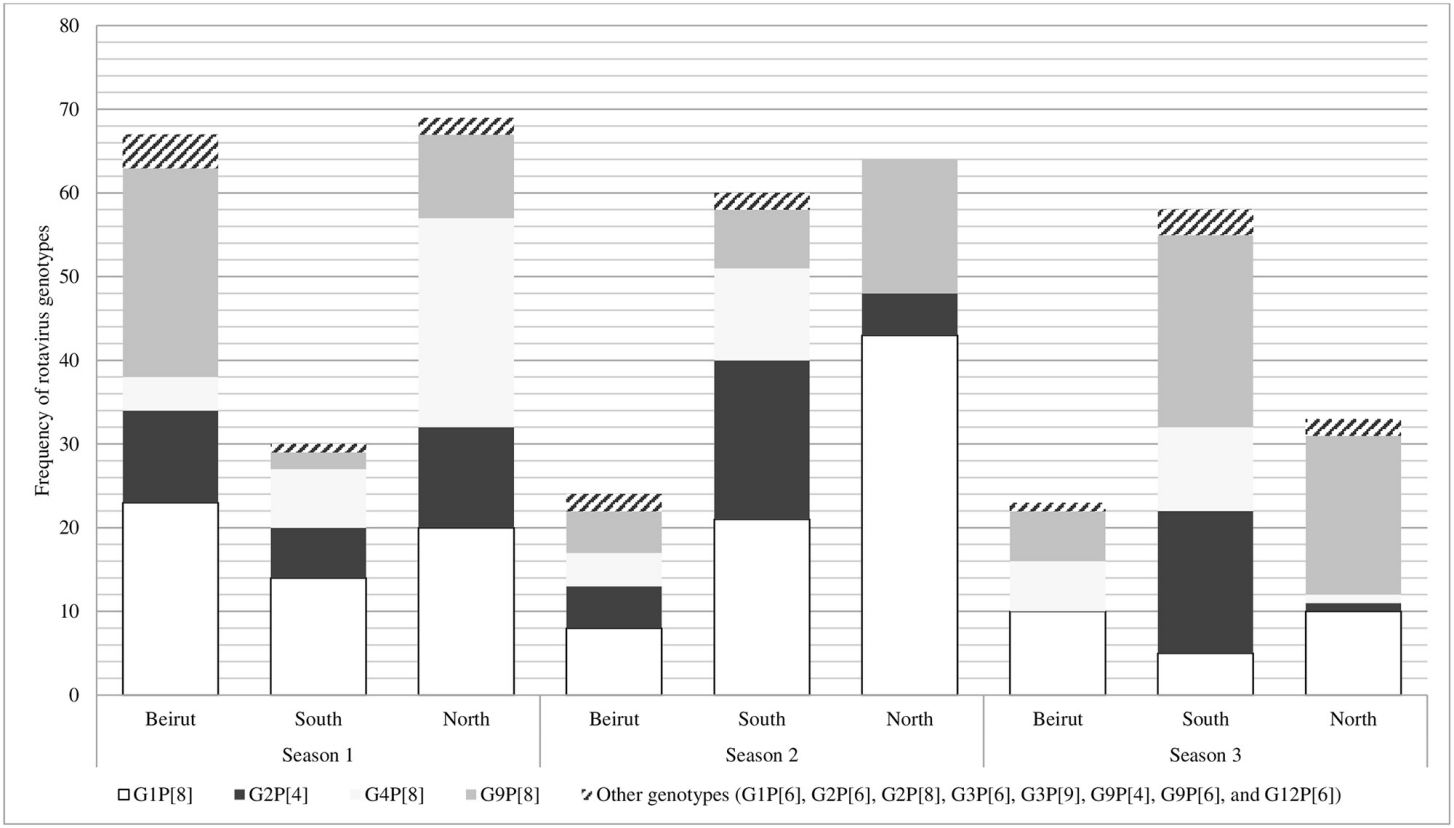
Distribution of rotavirus genotypes (in frequency) by season and geographical area of study center (n = 428). Geographical areas of study centers: Beirut: American University of Beirut Medical Center, Makassed Hospital and Rafic Hariri University Hospital; North: Nini Hospital; South: Hammoud Hospital and Nabatieh Hospital. Seasons: Season 1: January 2011 to August 2011, Season 2: September 2011 to August 2012, and Season 3: September2012 to June 2013.

RV genotypes were also different by season and across geographical areas of the study centers. In season 1, G9P[8] (n = 25, 16.4%) and G1P[8] (n = 23, 34.3%) dominated in Beirut, while the most common genotypes were G1P[8] (n = 14, 46.6%) in South Lebanon, G4P[8] (n = 25, 41.6%) and G1P[8] (n = 20, 29%) in North Lebanon. G4P[8] disappeared from the North in seasons 2 and 3 and G2P[4] did not circulate in Beirut in season 3 although it was strongly present in South Lebanon during the same season (Fig 3). Overall, G1P[8] was significantly predominant in North Lebanon (*P* = 0.007) all seasons combined, while G2P[4] predominated in the South *(P*<0.001). In parallel, G9P[8] was the most frequent genotype in North Lebanon with no significant predominance (*P* = 0.186). The same pattern applies for G4P[8] in the South (*P* = 0.344). Overall, and despite the small area of Lebanon, there was seasonal and geographic variation in the distribution of genotypes (Fig 3).

**Fig 4:**
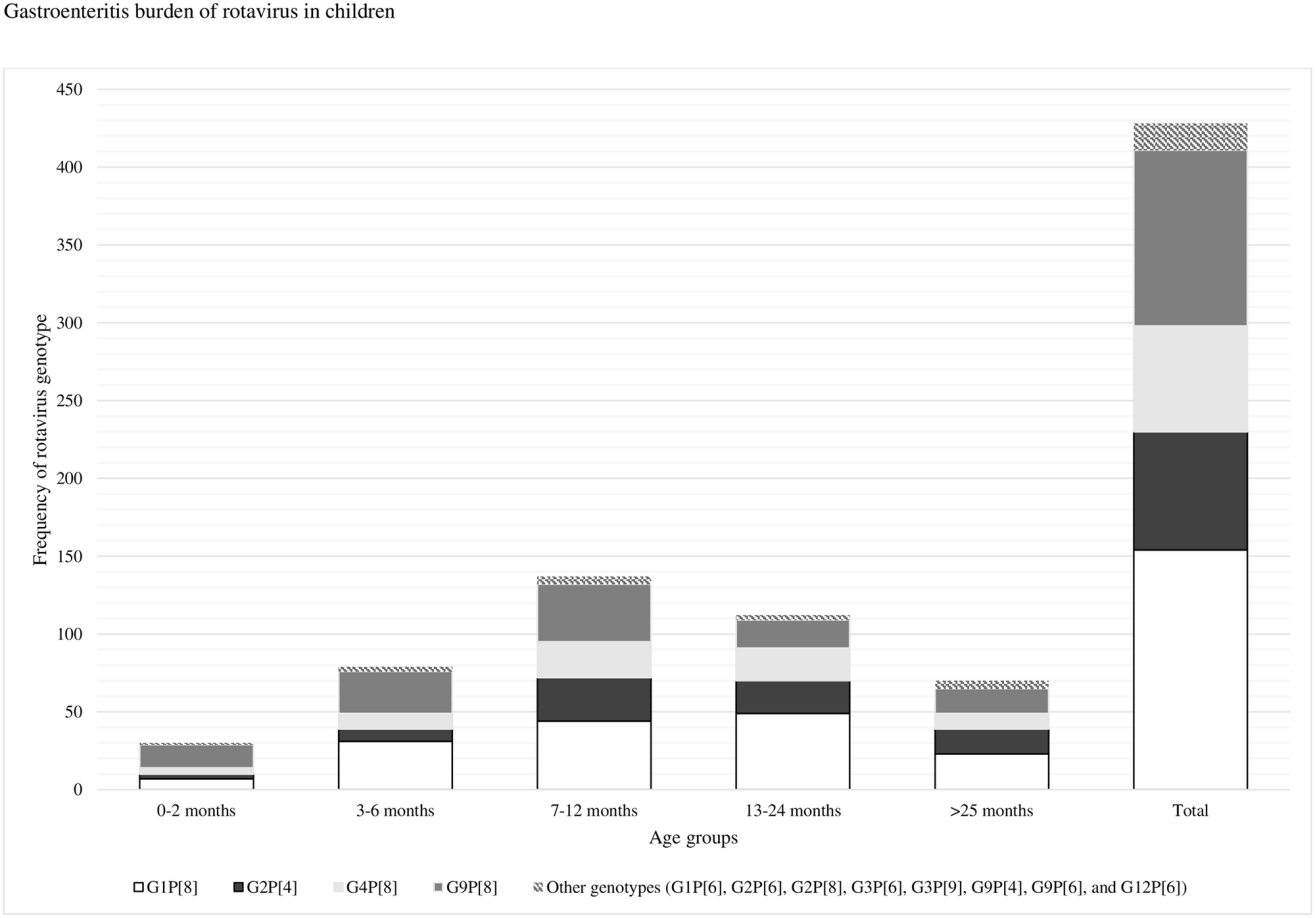
Distribution rotavirus genotypes by age group (n = 428).

All genotypes appeared equally in all age-groups examined (Fig 4). The Vesikari score did not differ significantly between the different genotypes (Table 2).

**Table 2:** Vesikari score for GE associated with the common rotavirus genotypes in vaccinated *versus* unvaccinated patients.

Predominant rotavirus genotype	Vaccinated	Unvaccinated	*P*-value*
	N	N	
	Mean ± standard deviation	Mean ± standard deviation	
**All genotypes**	48	378	0.339
	13.81 ± 2.70	14.19 ± 2.54	
**G1P[8]**	11	141	0.950
	14.45 ± 2.11	14.50 ± 2.50	
**G2P[4]**	15	61	0.420
	13.47 ± 2.77	13.89 ± 2.65	
**G4P[8]**	9	59	0.088
	12.56 ± 1.81	14.07 ± 2.52	
**G9P[8]**	11	102	0.696
	14.45 ± 3.42	14.03 ± 2.49	

*Significant at the 5% level.

### Vaccination status of patients diagnosed with GE and vaccine effectiveness

Only 18.1% (n = 255) of the children hospitalized for GE were vaccinated against rotavirus. Patients who were RV-negative were more likely to be vaccinated as compared to the RV-positive children (21% *versus* 11.3%, *P*<0.001). Moreover, 219 patients were vaccinated with RV1 and 28 with RV5 at a ratio of 7.8 RV1-vaccinated patient for each RV5-vaccinated patient. A 2-dose series of RV1 had been given to 6.4% (n = 27) of RV-positive patients *versus* 14.1% (n = 138) of RV-negative children (*P*<0.001) (Table 1).

Regardless of their RV status, the 255 vaccinated patients hospitalized for GE significantly differed from the 1,157 unvaccinated cohort by study center, age category and duration of hospitalization. The highest rate of vaccination (20.6%) was in the category of 13–24 months whereas, understandably, the highest rate of unvaccinated children diagnosed and hospitalized for GE was in the category of 0–2 months (*P* = 0.004). The rate of RV GE in the vaccinated cohort was significantly lower than the unvaccinated cohort (18.8% vs 32.7%, *P*<0.001). We reasoned that vaccinated patients might have lower disease severity due to at least partial protection provided by the vaccine. Nevertheless, when the Vesikari severity score was compared between vaccinated and unvaccinated patients, there was no statistical difference when analyzed as individual genotypes or as the total genotypes (Table 3).

**Table 3:** Vaccination status of patients diagnosed with gastroenteritis.

Variable	Vaccinated	Not Vaccinated	*P*-value
	(n = 255)	(n = 1,157)	
**Center**			<0.001*
AUBMC	61 (38.9%)	96 (61.1%)	<0.001*^£^
Hammoud Hospital	57 (27.5%)	150 (72.5%)	
Hôtel-Dieu de France University Hospital	3 (60.0%)	2 (40.0%)	
Makassed Hospital	18 (9.5%)	171 (90.5%)	
Nini Hospital	59 (10.6%)	497 (89.4%)	
Nabatieh Hospital	52 (24.5%)	160 (75.5%)	
Rafic Hariri University Hospital	5 (5.8%)	81 (94.2%)	
**Age (months)**			0.008*
0–2	4 (4.3%)	89 (95.7%)	
3–6	52 (19.4%)	216 (80.6%)	
7–12	84 (18.3%)	375 (81.7%)	
13–24	75 (20.6%)	289 (79.4%)	
25–60	40 (17.5%)	188 (82.5%)	
**Vesikari score**			0.866
≤10	22 (18.6%)	96 (81.4%)	
>10	233 (18.0%)	1,060 (82.0%)	
**Duration of hospitalization (days)**‡			0.001*
0–3	116 (45.5%)	420 (36.3%)	
4–7	135 (52.9%)	669 (57.8%)	
>7	4 (1.6%)	68 (5.9%)	
**Year**			0.308
2011	116 (17.0%)	567 (83.0%)	
2012	104 (20.1%)	413 (79.9%)	
2013	35 (16.5%)	177 (83.5%)	

*Significant at the 5% level.

^£^*P*-value excluding Hôtel-Dieu de France University Hospital from the comparison.

^‡^ Percentages are computed within each column.

Abbreviations: AUBMC = American University of Beirut Medical Center.

Forty eight cases of rotavirus infection occurred in vaccinated patients, leading to a vaccine breakthrough rate of 18.8%. Importantly, 40 out of 219 patients (18.3%) vaccinated with RV1 and 7 out of 27 patients (26%) vaccinated with RV5 had not completed the recommended vaccination schedule with the relevant vaccine. We then analyzed the RV genotype distribution of RV1 and RV5 breakthrough cases (Table 4).

**Table 4:** Vaccine breakthrough by vaccine type.

Predominant rotavirus genotype	Vaccine breakthrough n(%)		Vaccine breakthrough per number of doses n(%)				
	RV1 (Rotarix^®^)	RV5 (Rotateq^®^)	RV1 (Rotarix^®^)		RV5 (Rotateq^®^)		
			1 dose	2 doses	1 dose	2 doses	3 doses
**G1P[8]**	10 (25%)	1 (14.3%)	7 (17.5%)	3 (7.5%)	0 (0%)	0 (0%)	1 (14.3%)
**G2P[4]**	13 (32.5%)	1 (14.3%)	4 (10%)	9 (22.5%)	0 (0%)	0 (0%)	1 (14.3%)
**G2P[8]**	1 (2.5%)	-	0 (0%)	2 (2.5%)	-	-	-
**G4P[8]**	6 (15%)	3 (42.9%)	0 (0%)	6 (15%)	2 (28.6%)	0 (0%)	1 (14.3%)
**G9P[8]**	10 (25%)	1 (14.3%)	2 (5%)	8 (20%)	0 (0%)	0 (0%)	1 (14.3%)
**G9P[4]**	-	1 (14.3%)	-	-	0 (0%)	1 (14.3%)	0 (0%)

The majority of the genotypes where completely or partially homotypic with the vaccine types with the heterotypic exceptions being G2P[4] in RV1 (G1P[8]) vaccinated patient and G9P[4] in RV5 (G1,G2, G3, G4, and P[8]) vaccinated patient.

Vaccine effectiveness was computed for all vaccine types combined. Using RV-negative controls, the unadjusted VE was 62.7% (95%CI, 41.2%-76.3%). After adjustment by geographical area and season, VE against RV disease was 68.4% (95%CI, 49.6%-80.2%). Vaccine effectiveness was highest among the G1P[8] genotype; but for G2P[4] and G4P[8], VE was much lower with a wide confidence interval due to the small numbers (Table 5).

**Table 5:** Vaccine effectiveness against rotavirus disease.

	Vaccine Effectiveness (confidence interval at 95%)
**Unadjusted**	0.627 (0.412, 0.763)
**Adjusted for geographical area**	0.648 (0.443, 0.778)
**Adjusted for geographical area and season**	0.684 (0.496, 0.802)
**By geographical area**	
Beirut	0.647 (0.136, 0.855)
North	0.747 (0.273, 0.912)
South	0.598 (0.245, 0.786)
**By RV genotype**	
G1P[8]	0.921 (0.677, 0.981)
G9P[8]	0.587 (0.033, 0.824)
G2P[4]	0.209 (0.000, 0.617)
G4P[8]	0.415 (0.000, 0.753)

**Seasons:** Season 1: January 2011 to August 2011, Season 2: September 2011 to August 2012, and Season 3: September 2012 to June 2013.

**Geographical areas of study centers:** Beirut: American University of Beirut Medical Center, Makassed Hospital and Rafic Hariri University Hospital; North: Nini Hospital; South: Hammoud Hospital and Nabatieh Hospital.

## Discussion

To our knowledge, our study is the second epidemiological study, and the first extending over three rotavirus seasons, estimating the disease burden and the prevalent genotypes of rotavirus causing gastroenteritis in children based on a sentinel hospital-based surveillance program in Lebanon. Further, it evaluated the difference in clinical outcomes between the vaccinated and unvaccinated cohorts with GE, and provided data about the effectiveness of the two oral vaccines, RV1 and RV5 against RV disease in Lebanon.

### Socio-demographic and clinical characteristics of patients diagnosed with GE

Our analysis demonstrates that the burden of RV GE is significant in Lebanon with the rate of hospitalized GE caused by RV being 30.3% (n = 428, 95%CI, 27.9%-32.7%) between 2011 and 2013. This is lower than the findings reported in studies carried out in other developing countries in the Middle East. The percentage of rotavirus infection in hospitalized infants and young children was 37% in Iraqi Kurdistan [25], 39% in both Turkey [26,27] and Jordan [27], 40% in Kuwait [28], 45.2% in Yemen [29] and 49% in Oman [30]. The highest rate of rotavirus infection was recorded in Syria (61%) [27]. Contrarily, lower percentages of rotavirus infection were reported in our previous hospital-based surveillance study where GE attributable to RV was 27.7% [21]. Lower rates were also documented in Saudi Arabia (16%) [27] and Egypt (25.2%) [31]. Additionally, the rate of RV GE observed in this study was higher than those in developed countries such as the USA where RV was found to be responsible for 22% of childhood diarrheal hospitalizations [32].

These variations may reflect actual differences in RVGE rates but may also be related to variations in study design, the methods used in detecting rotavirus, and vaccination against RV. Indeed, high RV vaccination coverage rates were reported in the developed countries where mass vaccination programs have been implemented (72% in USA, 84%-87% in Australia, 87% in Austria and 85% in Belgium) leading to a significant drop in rates of RV-related hospitalizations [33]. In Lebanon, RV vaccination is currently not included in the national immunization program but is available in the private sector. The seven sentinel surveillance hospitals in our study serve families from a range of socioeconomic backgrounds, including those with very low to high income and this is reflected in the vaccination rates observed that ranged from 5.8% to 60%. Also, the present study found that, despite the use of vaccines, the rotavirus prevalence has not changed, but rather increased by a few points. This is likely due to suboptimal herd immunity as only 18.1% of subjects had received rotavirus vaccine.

As for age, 83.6% of RV GE was in children aged below two years. Our findings corroborate the results of our previous study conducted in Lebanon, in the same settings as the present study, where 75% of the RV GE cases occurred in children under two years of age [21]. Further, the difference between male and female in this study was not significant (P = 0.734), this result is concordant with previous studies [29,34].

Most (61.0%) of the 428 RV-positive patients required hospitalization for 4 to 7 days and 93% of them had high severity (Vesikari score >10). When compared with RV-negative patients, no statistically significant difference was found for the duration of hospitalization or the severity score. Many previous studies illustrated the burden of hospitalization due to RV GE. Hospitalization attributed to RV rose from 13.5% in the period 1999–2000 to 17.1% in the study period (2001–2005) in Spain in children aged less than 5 years old [35]. RV also accounted for 40% of diarrheal in-patient consultations in Argentina [36] and, in USA, RV led to 55,000 to 70,000 hospitalizations in children younger than 5 years of age in the post-vaccine era [37].

The significant difference between the RV-positive and RV-negative patients concerning fever and vomiting are not conclusive in the clinical diagnosis of RV GE, because such symptoms are not distinguishable from other types of GE. Nonetheless, the frequent combination of vomiting (62.9%) with diarrhea (91.1%) leads to the risk of dehydration in RV GE more than other types of GE and therefore increases the need for hospitalization and rehydration. A concerning finding in our study is that 40.4% of the patients with RV GE still received antibiotics, an unnecessary measure in a viral infection.

Over the 30-month-long surveillance period, the 1414 cases of GE occurred during all seasons but the majority was observed from December till March (41.1%), followed by July (12.3%). Also, RV GE was much more predominant in the cold season (69.9%). This is similar to the pattern seen in the literature including our previous study, which showed the same seasonal occurrence [21,27,35,38,39].

### RV genotyping

The most prevalent genotypes were G1P[8] (36%) followed by G9P[8] (26.4%), G2P[4] (17.8%) and G4P[8] (15.9%). Our most prevalent genotypes were recorded in a Yemeni study from 2014 where two of the major global human RV genotypes (G2P[4] 55% and G1P[8] 15%) were detected [29]. Our findings are also in agreement with a study from Iraq where G1P[8] (33%),G4P[8] (21%), G2P[4] (15%), G9P[8] (11%) and G1P[6] (11%) were the most frequently detected RV genotypes previously reported from Erbil in 2005 [24]. In Oman, the most frequent rotavirus genotypes in children aged less than 5 years and hospitalized for gastroenteritis, were G2P[4] (26%), G1P[8] (3%) and G1P[4] (3%) and G9P[4] (3%) [29]. Importantly, RV genotypes distribution in our study showed a different predominance profile across the three seasons, with G9P[8] being predominant in seasons 1 and 3, and G1P[8] in season 2 (*P*<0.001) (Fig 1). This may suggest that the same genotype is unlikely to predominate for two successive seasons due to the exhaustion of the susceptible infant population. A Brazilian study showed that G9P[8] was most prevalent in 2011 and G12P[8] in 2012 [40]. It was interesting to note that, despite the small area of Lebanon, there was significant geographic variation in the dominant genotypes. Whereas the four major genotypes circulated during each of the three seasons when all of Lebanon was considered, the relative predominance of specific genotypes varied between different regions. It is noteworthy that different genotypes prevailed in different countries, but the discrepancies with our findings could be due to differences in the year(s) covered in each study.

### Vaccination status of patients diagnosed with GE and vaccine effectiveness

The vaccination and RV rates were significantly correlated (Pearson’s chi-square coefficient of 19.1, *P*<0.001) as RV infections decreased with vaccinations. Also, the length of hospitalization for 4–7 days was significantly lower in the vaccinated patients (52.9%) compared to the unvaccinated ones (57.8%) (*P*<0.001) possibly indicating that vaccinated patients are able to recover faster. The vaccine breakthrough rate of 18.8% and the vaccine effectiveness of 68.4% observed in our study is commensurate with those of previous post-marketing studies assessing the effectiveness of the pentavalent (Rotateq^®^) and monovalent (Rotarix^®^) rotavirus vaccines in real-world settings [41–44]. Our study was not designed to measure differences in effectiveness between the vaccines. The majority of patients in our study were vaccinated with Rotarix^®^. Our VE results against RV disease are reassuring for a middle-income country like Lebanon. They also highlight the effectiveness of vaccination against RV and the importance of mass immunization to decrease its burden. Vaccination rate is still very low despite vaccine effectiveness, consequently the frequencies of RV cases did not change between the two studies [21].

### Study limitations and strengths

One limitation of our study is the inability to measure the true burden of rotavirus disease. The number of patients cared for by the different hospitals will be inaccurate due to the multiplicity of hospitals serving the same population. Nonetheless, this study is the first Lebanese and regional one to look at three consecutive seasons of rotavirus. This has implications regarding season-to-season variation in the predominant genotype and the relevance of vaccine used. Importantly, our study was also designed to evaluate both the burden of RV disease in hospitalized children and vaccine effectiveness of the 2-dose RV1 (Rotarix^®^) and 3-dose pentavalent vaccine (RV5, Rotateq^®^) series against rotavirus disease.

### Conclusion

In conclusion this study provides updated information on the extent of rotavirus-specific hospitalizations of RV GE, the prevalent genotypes of RV, vaccine effectiveness, and the impact of vaccination against the pathogen in Lebanon. Our results indicate that gastroenteritis caused by rotavirus in Lebanon is an important health problem, particularly among children aged under two years, mostly during the winter season. Also, our findings provide compelling evidence of the potential benefits of RV vaccination among infants and young children with acceptable VE. However, the data provided show a low rate of vaccination (18.1%), despite the availability of the two oral vaccines RV1 and RV5 in Lebanon but only in the private sector. Therefore, government supported vaccinations are of utmost importance to ensure mass immunization. The present results would help inform the health policymakers about the RV GE burden and underscore the need for national immunization programs to be implemented all over the country.

## Supporting Information

S1 CRF Case Report Form (CRF)—Rotavirus study. (PDF)
