# Rotavirus Genotypes and Vaccine Effectiveness from a Sentinel, Hospital-Based, Surveillance Study for Three Consecutive Rotavirus Seasons in Lebanon

**DOI:** 10.1371/journal.pone.0286701

**Published:** 2023-06-02

**Authors:** Zainab Ali, Houda Harastani, Moza Hammadi, Lina Reslan, Soha Ghanem, Farah Hajar, Ahmad Sabra, Amjad Haidar, Adlette Inati, Mariam Rajab, Hassan Fakhouri, Bassam Ghanem, Ghassan Baasiri, Bernard Gerbaka, Hassan Zaraket, Ghassan M. Matar, Ghassan Dbaibo

In Figs 1, 2, 3, and 4 the genotype labels are incorrect. Please see the corrected versions here.

**Fig 1:**
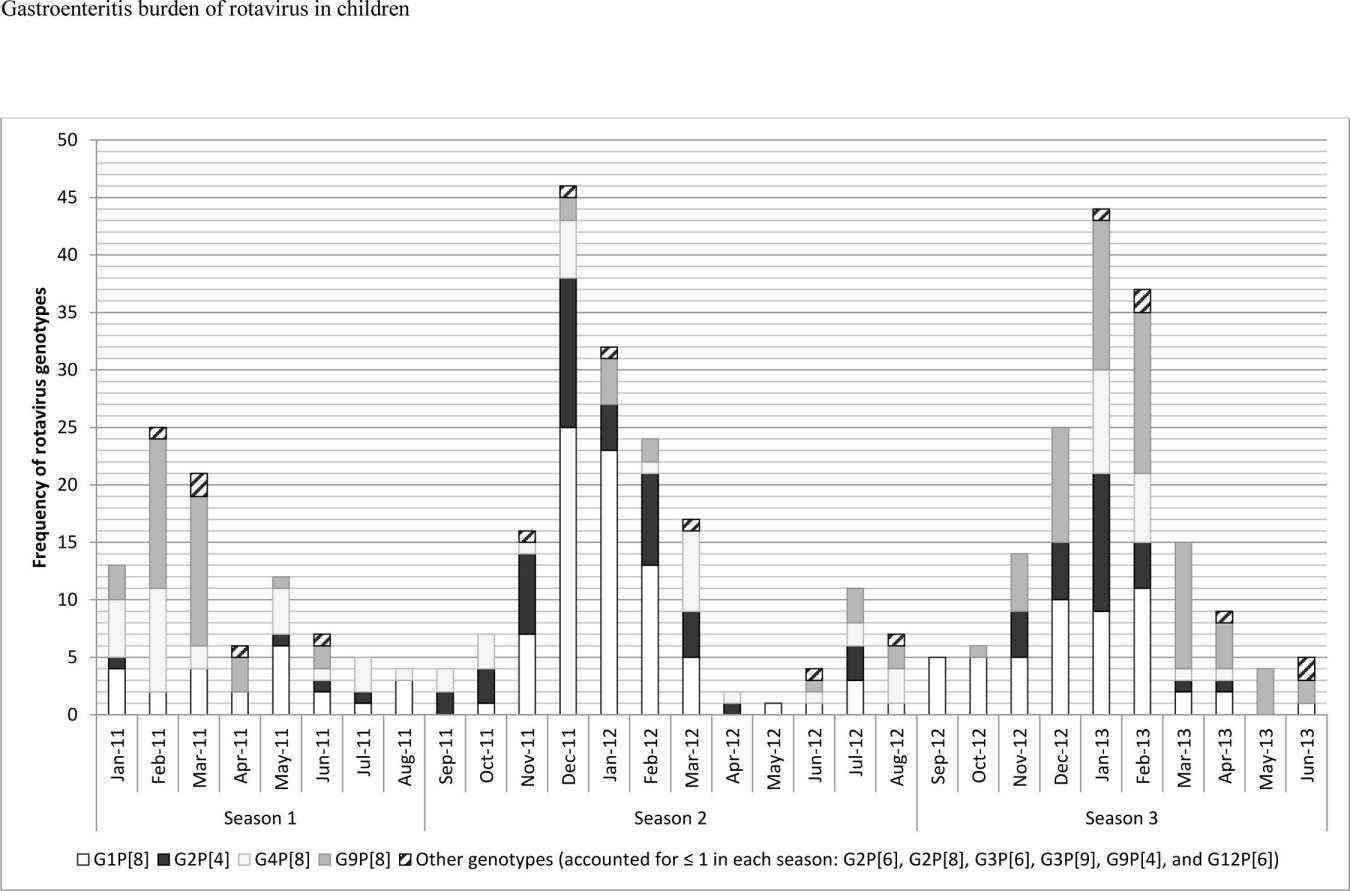


**Fig 2:**
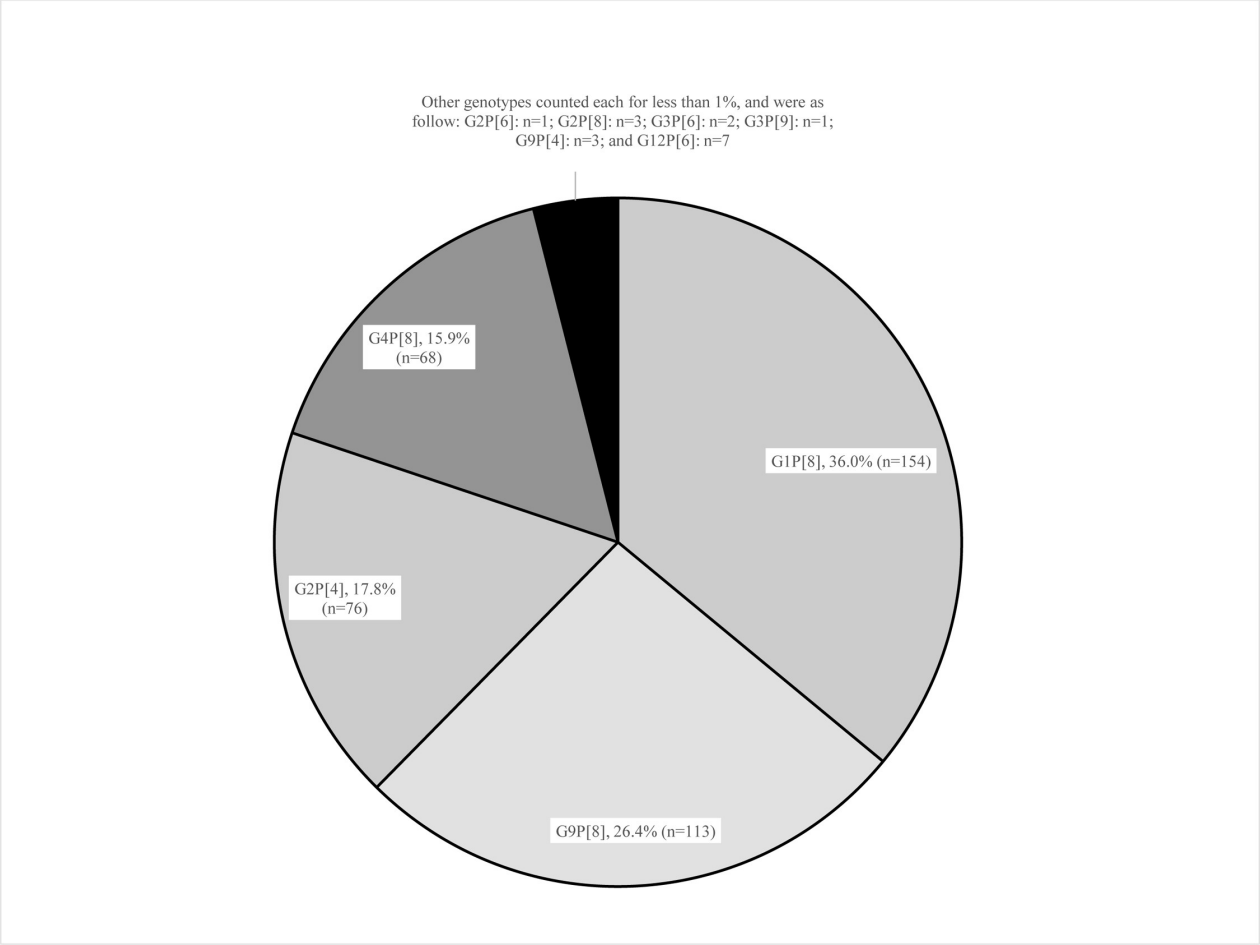


**Fig 3:**
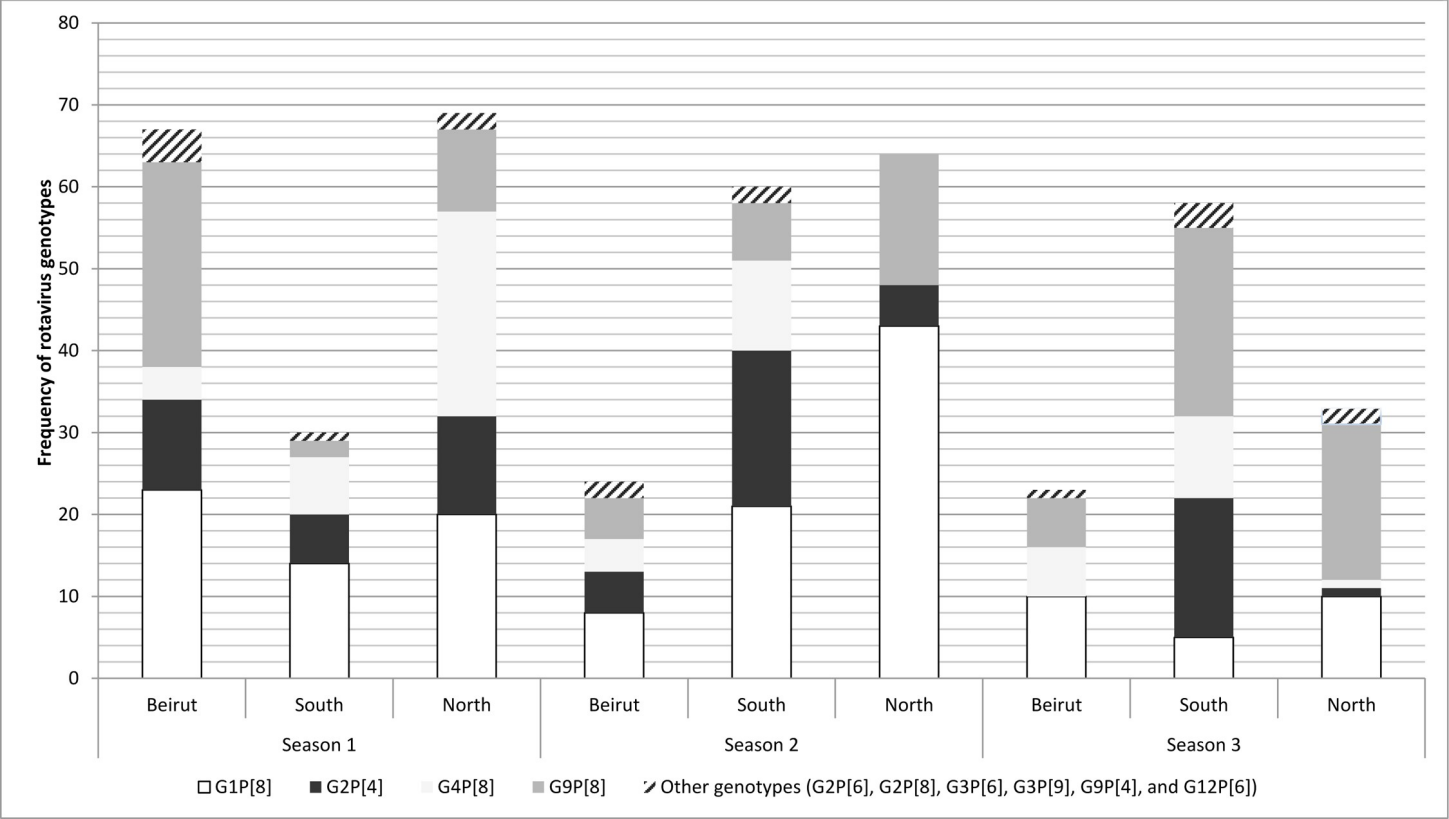


**Fig 4:**
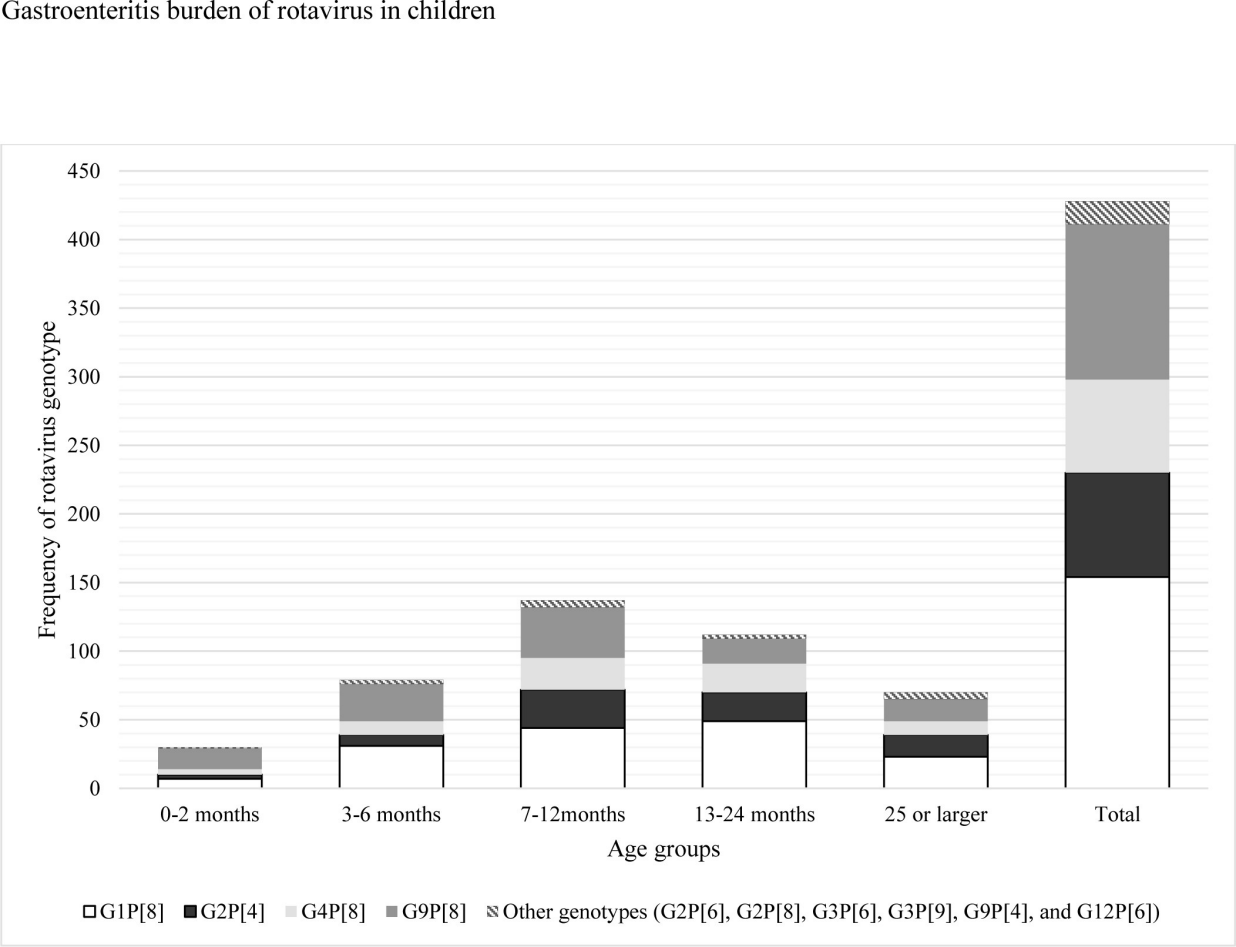


## References

[1] Ali Z, Harastani H, Hammadi M, Reslan L, Ghanem S, et al. (2016) Rotavirus Genotypes and Vaccine Effectiveness from a Sentinel, Hospital-Based, Surveillance Study for Three Consecutive Rotavirus Seasons in Lebanon. PLOS ONE 11(8): e0161345. doi: 10.1371/journal.pone.0161345 27571515 PMC5003350

