# Supplementary material for: Rotavirus Genotypes and Vaccine Effectiveness from a Sentinel, Hospital-Based, Surveillance Study for Three Consecutive Rotavirus Seasons in Lebanon
Source: PLoS One. 2016 Aug 29;11(8):e0161345. doi: 10.1371/journal.pone.0161345 (PMC5003350; doi:10.1371/journal.pone.0161345)
Supplement: S1 CRF — (PDF) [file pone.0161345.s001.pdf]

Date of Interview: ____________ Subject Number:__________

Rotavirus Study

Case Report Form

Center:

Estimation of the disease burden and prevalent genotypes of rotavirus (RV) causing gastroenteritis (GE) in children < 5 years of age in Lebanon based on a sentinel hospital-based surveillance program.

Date of Interview: ____________ Subject Number:__________

Informed consent

I certify that the informed consent had been obtained prior to any study procedure.

Date of informed consent (Day/ month / year): _____ _____ _____

Center Name: ____

Date of Birth (Day/Month/year): _____ _____ _____

Gender: ___ Male ___ Female

Height: _________ (cm)

Weight: _________(kg)

Does the subject live in Lebanon? ___ Yes ____ No

GENERAL TREATMENT FOR GE EPISODE BEFORE HOSPITALIZATION
Note: do not include treatment for GE on the day of hospitalization here!!!

	**Yes**	**No**	**Unknown**
**Oral rehydration therapy:**	___	___	____
**IV rehydration therapy:**	___	___	____
**Antibiotics:**	___	___	____

	YES	NO	UNKNOWN
**Rotavirus vaccinated**	____	____	____

**If yes**	**Dates**	**Dates**	**Dates**
**ROTARIX**	___/___/___	___/___/___	
**ROTATEQ**	___/___/___	___/___/___	___/___/___

PREVIOUS STUDY PARTICIPATION

Has the subject previously participated in this study? _____ Yes _____ No

<u>TO BE COMPLETED AT THE HOSPITAL SITE</u>

ELIGIBILITY QUESTION

Did the subject meet all the entry criteria?

_____ Yes ______ No → If No, tick all boxes corresponding to violations of any inclusion/exclusion criteria

INCLUSION CRITERIA

Tick the boxes corresponding to any of the inclusion criteria the subject failed.

_____ Subjects for whom the investigator believes that their parents/guardians can and will comply with the requirements of the protocol should be enrolled in the study.

_____ A male or female child <5 years of age at the time of admission to the study hospital or its Emergence Department for GE (a child becomes ineligible on the day of his/her fifth birthday)

_____ Written informed consent obtained from the parents/guardians of the subject.

EXCLUSION CRITERIA
Tick the boxes corresponding to any of the exclusion criteria that disqualified the subject from entry.

_____ The diagnosis for treatment at the study site does not include GE

_____ The onset of GE after admission to the hospital i.e. within 12 hours after hospital admission (nosocomial infections).

Date of Admission (Day/Month/Year): ____

Diagnosis at Admission: _____ Gastroenteritis

_____ Other ____

_____ Unknown

Date of Interview: ____________ Subject Number:__________

GENERAL SYMPTOMS OF GE EPISODE <u>BEFORE</u> HOSPITALIZATION
Symptoms of GE on the day of hospitalization are not included here

**Fever**	**Yes**	**No**	Unknown	
	_____	_____	_____	**If yes** → _ _ _ days with fever **before** hospitalization:
≥ 37.5ºC Axillary				→Maximum temperature: _ _ _ _ _ ºC
≥38.0ºC Rectal or 38.0ºC Tympanic				→Maximum temperature measured by:<br>____ Axillary<br>____ Rectal<br>____ Tympanic

**Vomiting**	Yes	No	Unknown	
Forceful emptying of partially digested stomach contents ≥ 1 hour after feeding	_____	_____	_____	_ _ _ days of vomiting **before** hospitalization
				→ Maximum # of emeses per day:<br>__1 __ 2 __3 __4 __5 __6 __ ≥8

**Diarrhea:**	Yes	No	Unknown	
3 or more looser than normal stools within "24h"	_____	_____	_____	_ _ _ days of diarrhea **before** hospitalization
				→ Maximum # of diarrheal stools per day::<br>__1 __ 2 __3 __4 __5 __6 __ ≥8

	___ No dehydration	___ Severe (≥ 6%)

Date of Interview: ____________ Subject Number:__________

**Dehydration:**	___ Mild/Moderate (1 to 5 %)	___ Unknown

→To be answered by an MD
→According to your clinical assessment, what was the child's degree of dehydration at admission?

DURING THE HOSPITALIZATION AND AT DISCHARGE

Treatment	Yes	No	Unknown
Oral rehydration therapy:	____	____	____
IV rehydration therapy:	____	____	____
Antibiotics:	____	____	____

Please check by a cross(X) the exact box

STOOL SAMPLE

Question	Unknown	No	Yes	If Yes
**Has a stool sample been collected?**	____	____	____	Please provide a date (d/m/y) ___ ____ ___
**Has the stool sample been tested for RV?**	____	____	____	RV (-) ____<br>RV(+) ____
**Has the RV Positive stool sample been serotyped for rotavirus?**	____	____	____	G1 ___<br>G2 ___<br>G3 ___<br>G4 ___<br>G9 ___<br>Untypable ____<br>Other ________

Date of discharge (Day/month/year): _____ _____ _____

Weight at discharge: ____________________________ (Kg)

Date of Interview: ____________ Subject Number:__________

Diagnosis at discharge:

___ Gastroenteritis

___ Other ____

___ Unknown

GENERAL SYMPTOMS OF GE EPISODE DURING HOSPITALIZATION

**Fever**	Yes	No	Unknown	
	_____	_____	_____	**If yes** → _ _ _ days with fever **during** hospitalization:
≥ 37.5ºC Axillary				→Maximum temperature: _ _ _ _ _ ºC
≥38.0ºC Rectal or 38.0ºC Tympanic				→Maximum temperature measured by:<br>____ Axillary<br>____ Rectal<br>____ Tympanic

**Vomiting**	Yes	No	Unknown	
Forceful emptying of partially digested stomach contents ≥ 1 hour after feeding	_____	_____	_____	_ _ _ days of vomiting **during** hospitalization
				→ Maximum # of emeses per day:<br>__1 __ 2 __3 __4 __5 __6 __ ≥8

**Diarrhea:**	Yes	No	Unknown	
3 or more looser than normal stools within "24h"	_____	_____	_____	_ _ _ days of diarrhea **during** hospitalization
				→ Maximum # of diarrheal stools per day::<br>__1 __ 2 __3 __4 __5 __6 __ ≥8

Date of Interview: ____________ Subject Number:__________

**Dehydration:**	___ No dehydration	___ Severe (≥ 6%)
	___ Mild/Moderate (1 to 5 %)	___ Unknown

→To be answered by an MD

→According to your clinical assessment, what was the child's degree of dehydration at admission?

OUTCOME OF THE GASTROENTERITIS AT DISCHARGE:

1: __ Recovered

2: __ Recovered with sequelae

3: __ Ongoing: → If ongoing, will active ambulatory follow-up be provided? __ Yes
__ No

4: __ Patient transferred to another hospital

5: __ Died → Date of death (Day / month / year) _ _ _ _/_ _ _ _/ _ _ _ _

6: __ Unknown

STUDY CONCLUSION
INVESTIGATOR'S SIGNATURE

I confirm that I have reviewed the data in this Case Report Form for this subject. All information entered by myself or my colleagues is, to the best of my knowledge, complete and accurate, as of the date below.

Investigator's signature: ________________________

Date (day/month/year) _ _ _ _/_ _ _ _/ _ _ _ _

The Vesikari 20 point scoring system

Vesikari et al. scale to assess **GE intensity**

Adverse experience	Points	Adverse experience	Points
**Duration of looser than normal stools (days)**		**Maximum number of episodes of Vomiting/24 hours**	
**1-4**	**1**	**1**	**1**
**5**	**2**	**2-4**	**2**
**≥6**	**3**	**≥5**	**3**
**Maximum number of looser than normal stools/24 hours**		**Fever measured rectally* (axillary)**	
**1-3**	**1**	**37.1-38.4°C** (36.6-37.9°C)	**1**
**4-5**	**2**	**38.5-38.9 °C** (38-38.4°C)	**2**
**≥6**	**3**	**≥39°C**	**3**
**Duration of vomiting (days)**		**Treatment**	
**1**	**1**	**Rehydration**	**1**
**2**	**2**	**Hospitalization**	**2**

**≥3**	**3**	**Dehydration**	
		**1-5%**	**2**
**TOTAL SCORE: ______**		**≥6%**	**3**

A score of 1-10 was considered as mild/moderate and a score ≥ 11 was considered as severe.

Reference:

Ruuska T, Vesikari T. Rotavirus disease in Finnish children: Use of numerical scores for clinical severity of diarreheal episodes. *Scand J Infect Dis. 1990; 22:259-267*
